# Identification of *FGF14* GAA Expansions in Polish Patients with Undiagnosed Cerebellar Ataxia – A Preliminary Study

**DOI:** 10.1007/s12311-026-02003-4

**Published:** 2026-05-07

**Authors:** Marta Matlawska, Karolina Ziora-Jakutowicz, Marie- Josee Dicaire, Joanna Pera, David Pellerin, Bernard Brais, Pablo Iruzubieta, Ewelina Elert- Dobkowska, Anna Sulek

**Affiliations:** 1 Department of Genetics, Institute of Psychiatry and Neurology, Sobieskiego 9 , 02-957 Warsaw, Poland; 2 Department of Neurology and Neurosurgery, Montreal Neurological Institute- Hospital, McGill University, Montreal, Québec Canada; 3 Department of Neurology, Jagiellonian University Medical College, Krakow, Poland; 4 Department of Neuromuscular Diseases, UCL Queen Square Institute of Neurology London and The National Hospital for Neurology and Neurosurgery, University College London, London, United Kingdom; 5 Faculty of Medicine, Lazarski University, Warsaw, Poland

**Keywords:** SCA27B, *FGF14* expansion, Spinocerebellar ataxia, Neurodegenerative disorders

## Abstract

Spinocerebellar ataxia type 27B (SCA27B), caused by an intronic GAA repeat expansion in the *FGF14* gene, has recently emerged as a major cause of late-onset cerebellar ataxia (LOCA). Its prevalence and clinical profile in Central and Eastern Europe remain largely unknown. To determine the frequency and phenotypic characteristics of *FGF14* GAA·TTC repeat expansions, a large cohort of Polish patients with undiagnosed adult-onset cerebellar ataxia was investigated. We retrospectively analyzed 701 patients (age of onset ≥ 25 years) with adult-onset cerebellar ataxia of unknown etiology, previously tested negative for SCA1, SCA2, SCA3, SCA8, and *RFC1* expansions. GAA·TTC repeat lengths were assessed using long-range and repeat-primed PCR. Expansions ≥ 250 repeats were classified as pathogenic. Clinical and MRI data were evaluated where available. A control group of 66 neurologically healthy individuals was also screened. Pathogenic *FGF14* expansions (≥ 250 repeats) were identified in 4.4% (31/701) of patients, including 23 with fully penetrant (≥ 300) and 8 with incompletely penetrant (250–299) alleles. No pathogenic expansions were found in controls. The mean age of onset was 49.8 years. Common symptoms included balance and gait disturbances, cerebellar syndrome, and episodic features such as diplopia. Cerebellar atrophy was present in 45% of patients with available MRI. No significant correlation between repeat length and age of onset was observed. Our findings confirm that *FGF14* repeat expansions are an underrecognized cause of LOCA in Poland and support their inclusion in standard genetic testing for adult-onset ataxias. Further studies are warranted to better define penetrance and genotype–phenotype correlations.

**Supplementary Information:**

The online version contains supplementary material available at 10.1007/s12311-026-02003-4.

## Introduction

Autosomal dominant spinocerebellar ataxias (SCAs) are a genetically and clinically heterogeneous group of neurodegenerative disorders characterized by progressive degeneration of cerebellar neurons. Despite continuous advancements in diagnostic tools, including sequencing technologies, the molecular basis of SCA remains unknown in a significant proportion of patients. An intronic GAA·TTC repeat expansion in the *FGF14* gene has recently been described as the cause of autosomal dominant spinocerebellar ataxia 27B (SCA27B) [1, 2, 3]. It is currently assumed that expansions of 250–299 GAA·TTC repeats are pathogenic, yet incompletely penetrant, while larger expansions are deemed pathogenic and highly penetrant [2, 4]. Studies involving different cohorts of patients with late-onset cerebellar ataxia (LOCA, AOO < 30 years) of unknown etiology have shown that SCA27B accounts for 10 to 60% of cases [1, 2]. The pathogenic threshold and phenotypic spectrum of SCA27B still remain to be fully characterized. The classic phenotype of SCA27B includes gait ataxia, downbeat nystagmus (DBN), and the occurrence of episodic symptoms [2, 5]. It is postulated that SCA27B is currently the most frequently identified type of autosomal dominant ataxia among patients in whom the genetic cause of another LOCA could not yet be determined.

In this paper, we present the results of our preliminary study on the assessment of the frequency of SCA27B in a cohort of Polish patients with spinocerebellar ataxia of unknown etiology.

## Methods

### Patients and Clinical Data

Inclusion criteria for the study comprised adult- onset cerebellar ataxia (AOO ≥ 25 years, range 25–83 years) of unknown etiology, with negative test results for SCA1, SCA2, SCA3, SCA8, and *RFC1*-related ataxia. A group of 701 patients (344 females and 357 males) with LOCA of unknown etiology was recruited retrospectively from patients referred to the Genetic Clinic of the Institute of Psychiatry and Neurology from all over Poland. Cases of MSA-C (Multiple System Atrophy- Cerebellar Type) were excluded from this study. The control group consisted of 66 healthy individuals (33 females and 33 males) without neurological symptoms, and the mean age was 69 years. In the patient’s group, 11/31 individuals with an *FGF14* repeat expansion had previously undergone brain MRI. Clinical data were collected retrospectively from available medical records. Imbalance was defined as subjective or examiner-reported postural instability without explicit documentation of cerebellar signs. Gait ataxia was recorded when a cerebellar gait disturbance was clearly described. Cerebellar syndrome was noted when the medical records documented a combination of cerebellar signs, including gait ataxia, appendicular (upper limb) ataxia, cerebellar dysarthria, and/or oculomotor abnormalities. In some cases, the diagnosis of *ataxia* was documented without detailed phenotypic characterization. These cases were recorded as *ataxia (unspecified)* and analyzed separately from gait ataxia, appendicular ataxia, and cerebellar syndrome, in order to preserve the original clinical documentation and avoid retrospective reclassification.

**Fig. 1:**
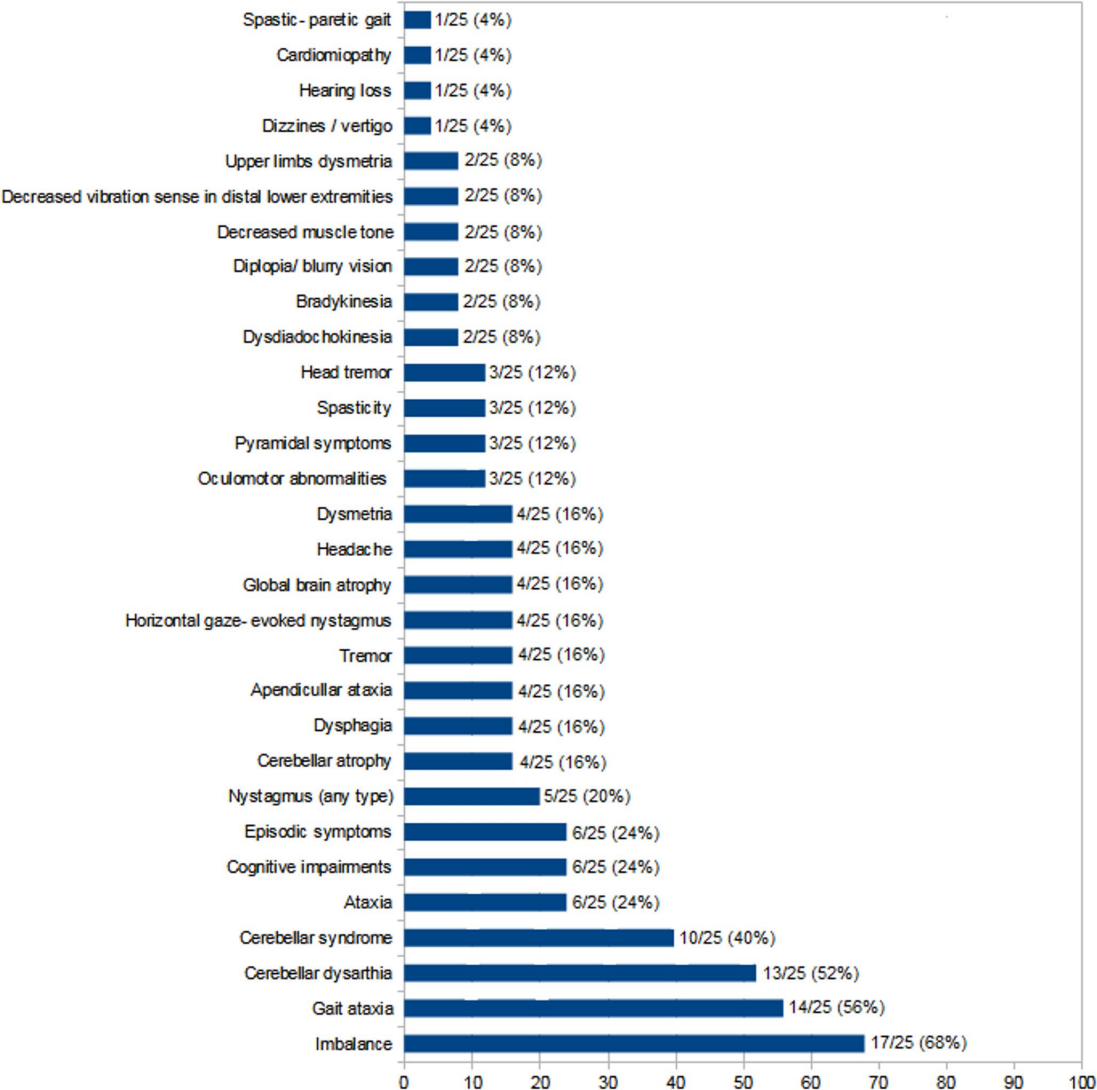
Frequency [%] of neurological features in 25 patients with available clinical data and confirmed pathogenic *FGF14* GAA·TTC repeat expansions (≥250 repeats). Percentages represent the proportion of patients presenting a given feature; individual patients could contribute to more than one category. Clinical features were categorized based on terminology used in the medical records

Speech disturbances described as *dysarthria*, *slurred speech*, or *scanning speech* were grouped under a single category of cerebellar dysarthria, reflecting overlapping manifestations of cerebellar speech involvement. When explicitly stated, appendicular ataxia was recorded separately. Due to the retrospective design of the study and heterogeneity of clinical documentation, not all neurological features could be assessed uniformly across patients. The results of clinical examination are shown in Fig. 1. Informed written consent to genetic testing was obtained from all participants, with a consent to participate in scientific research. The study was conducted in accordance with the Declaration of Helsinki and its subsequent amendments. The protocol was approved by the Local Bioethics Committee at the Institute of Psychiatry and Neurology in Warsaw. Informed consent was obtained from all individual participants included in the study.

### Molecular Examinations

**Fig. 2:**
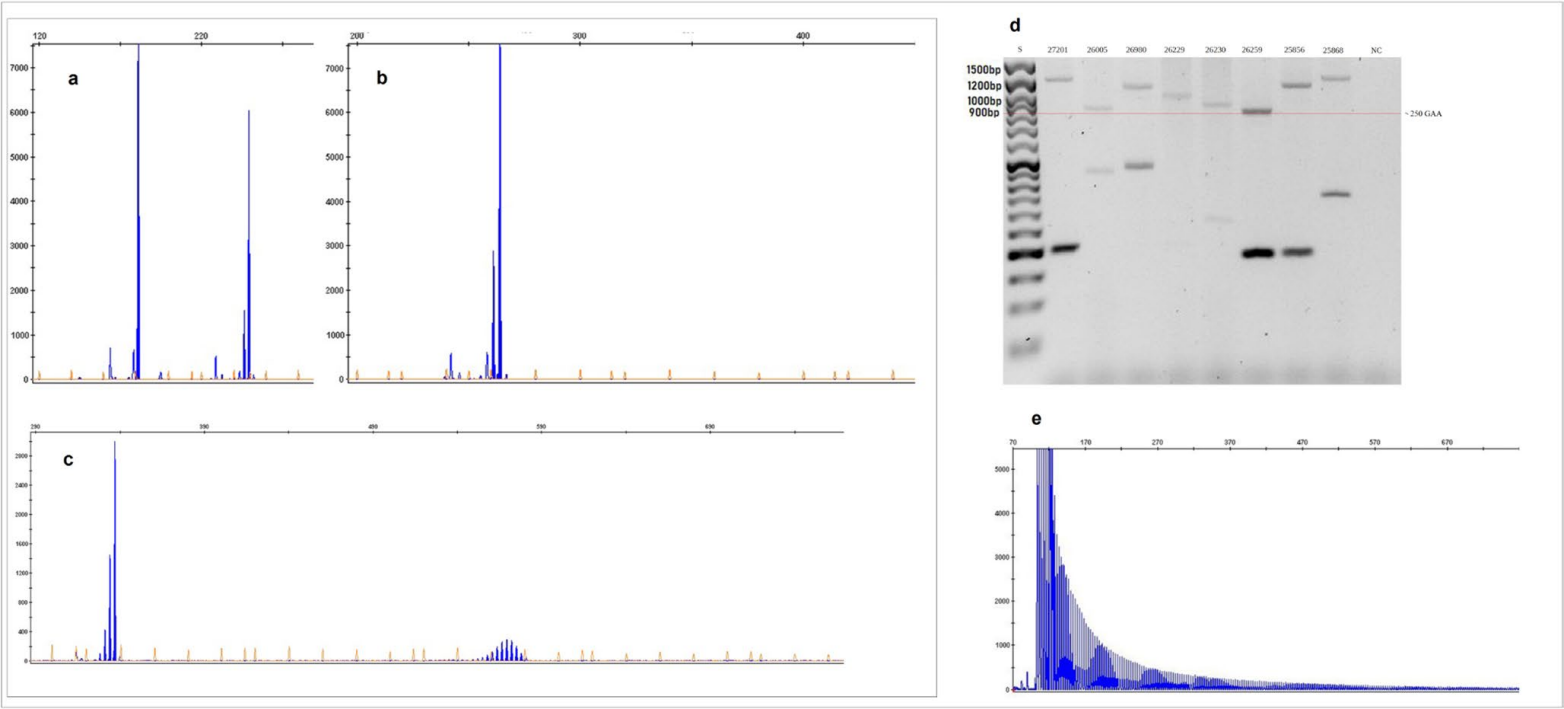
Molecular analysis of the *FGF14* GAA repetitive locus: electropherograms of the amplified *FGF14* repetitive locus using long- range PCR : **a** Heterozygote 10/33 GAA; **b** Homozygote 38/38 GAA; **c** Heterozygote 62/140 GAA with nonpathogenic expansion on second allele, **d** Agarose gel electrophoresis of LR-PCR amplicons with ≥250 GAA (900bp) on the expanded allele: S – size standard; NC – negative control, **e** Electropherograms of the amplified *FGF14* repetitive locus using repeat - primed PCR: characteristic sawtooth profile for patient with ≥250 GAA expansion

The GAA·TTC expansions were studied according to the standard protocol [4]. Expansions larger than 250 GAA·TTC repeats were considered pathogenic (Fig. 2, see also Figure S2-S6 for uncropped blots.) Molecular studies were performed at the Department of Genetics of the Institute of Psychiatry and Neurology in Poland and the Montreal Neurological Institute-Hospital – Mcgill University in Canada.

### Statistical Analysis

Statistical analyses were performed in R 4.5.0 and Rstudio software. Pearson’s coefficients were used to determine the correlation between the age of onset (AOO) and the number of GAA repeats, assuming statistically significant values when *p* < 0.05.

## Results

We examined 701 probands with adult- onset cerebellar ataxia. Positive family history of ataxia was found in 172 patients, while 529 patients were sporadic cases. Their mean age at examination was 60,5 years. Among them, 3.3% (23/701) had a confirmed GAA·TTC repeat expansion in *FGF14* within the fully penetrant range (301–583 repeats). An additional 1.1% (8/701) had expansions in the incompletely penetrant range (250–299 repeats). In total, we found 31 individuals (4.4%) carrying an *FGF14* repeat expansion larger than 250 GAA·TTC repeat units. The median size of the expanded allele was 345 GAA·TTC repeats. We also identified 2 patients carrying a non-pathogenic GAAGGA expansion. Among the 31 identified patients with SCA27B, 11 cases were familial (Figure S1, Suplemmentary materials), 14 had no family history, and this information was not available for 6 patients. The mean age of onset in patients with confirmed SCA27B was 49.8 (range 34–79 years). One of the patients carried biallelic expansions (287/383 GAA·TTC repeats) and his age of onset was 60 years. Based on the available clinical data at the time of evaluation, including age at onset, the clinical presentation did not appear markedly different from that observed in patients with a single pathogenic expansion. However, longitudinal clinical data and MRI results were not available for this patient. Due to the lack of SARA scale data, we were unable to assess disease progression. In our study, 4 patients with an intermediate allele, i.e. 200–249 GAA·TTC repeats (9/224, 9/232, 100/237 and 38/244 GAA·TTC), presented ataxia, including balance disturbances (4/4), gait disturbances (3/4), dysarthria (1/4), cerebellar atrophy and generalized brain atrophy (2/4). However, apart from excluding SCA1, SCA2, SCA3 and SCA8, we did not have the results of more detailed studies (e.g. WES, WGS) excluding other types of SCA in the indicated patients. In the control group, no alleles larger than 187 GAA·TTC repeats were identified (range 8–187 GAA·TTC). Our study demonstrated no significant inverse correlation between the number of GAA·TTC repeats and the age of onset in 25 patients (Pearson coefficient: *r* = -0.342, *p* = 0.087).

Detailed clinical information was available for 25 of the 31 patients with confirmed pathogenic *FGF14* GAA·TTC repeat expansions (≥ 250 repeats). Imbalance, defined as subjective or examiner-reported postural instability without explicitly documented cerebellar signs, was the most frequently reported symptom, observed in 68% of patients (17/25). Gait ataxia, referring to a clinically evident cerebellar gait disturbance, was present in 56% (14/25). A cerebellar syndrome, defined as the presence of multiple cerebellar signs documented by the examining neurologist, was reported in 40% of patients (10/25).

Among patients classified as having a cerebellar syndrome, gait involvement was documented in the majority of cases, while appendicular (upper limb) ataxia was reported when explicitly stated in the medical records. Due to the retrospective nature of the study and heterogeneity of clinical documentation, a precise topographical classification of cerebellar ataxia (gait versus limb involvement) was not uniformly available for all patients.

Speech disturbances were documented in 52% of patients (13/25) and were classified as cerebellar dysarthria, encompassing descriptions of dysarthria, slurred speech, and scanning speech used interchangeably in the medical records. Other neurological features are summarized in Fig. 1. Ataxia (unspecified), cognitive impairment, and episodic symptoms were each independently present in 24% (6/25) of the patients. Other frequent findings included: nystagmus (any type): 20% (5/25), cerebellar atrophy: 16% (4/25), appendicular ataxia, dysphagia, tremor, horizontal gaze-evoked nystagmus, global brain atrophy, headache, and dysmetria: each independently observed in 16% (4/25). Oculomotor abnormalities, pyramidal symptoms, spasticity, and head tremor: each independently found in 12% (3/25). Less commonly reported symptoms (8%, 2/25) included: dysdiadochokinesia, choking, bradykinesia, diplopia or blurry vision, decreased muscle tension, proprioceptive sensory disturbances, and upper limb dysmetria. Rare findings, each observed in a single patient (4%, 1/25), were dizziness/vertigo, hearing loss, cardiomyopathy, and spastic-paretic gait. These results indicate a predominance of core cerebellar features, particularly imbalance and gait disturbance, alongside a range of additional motor, sensory, and episodic symptoms in patients with *FGF14*-related ataxia.

## Discussion

Autosomal dominant SCA27B is a late-onset ataxia, commonly preceded by an episodic onset, with the onset of permanent symptoms often occurring in the fifth to seventh decade of life [2, 5]. In our cohort, the median age at onset was 50 years (30–79 years). The clinical phenotype in Polish patients with SCA27B is similar to that of other published cohorts, with a predominance of gait ataxia, horizontal-gaze evoked nystagmus, dysarthria, and episodic symptoms [1, 2, 5–15]. We found no significant correlation between the number of GAA repeats and the age of onset, although we observed a trend.

According to the previous epidemiological data, the highest percentage of patients with confirmed SCA27B (61%) is noted in the French-Canadian population, which is related to a founder effect [2].

**Table 1:** Number of patients (familial and sporadic cases) diagnosed with SCA27B in different ethnicity cohorts

Cohort ethnicity	No. of patients diagnosed with SCA27B (≥ 250 GAA·TTC)	References:
French Canadian	40/66 (61%)	[2]
Spanish	18/107 (28.1%)	[17]
German	34/148 (23%)	[15]
Australian	3/20 (15%)	[2]
Greek	19/160 (11.9%)	[8]
Italian	53/396 (13.4%)	[11]
Dutch	28/248(11%)	[12]
Brasilian	8/93 (9%)	[6]
Cyprus	12/155 (7.7%)	[9]
USA	55/732 (7.5%)	[14]
Serbian	9/167 (5.4%)	[13]
Northern Finnish	5/96 (5.2%)	[18]
Jewish	2/91 (2.2%)	[7]
Japanese	8/460 (1.7%)	[19]
Chinese	12/1216 (1.0%)	[20]

The first case of SCA27B in a Polish Family was reported in 2025 by Hirschfeld and colleagues [16]. In our cohort of 701 Polish patients with spinocerebellar ataxia of unknown cause, 31 cases carried a repeat expansion in *FGF14* gene, representing approximately 4.4%. Our results indicate that Poland currently has one of the lowest percentage of patients with SCA27B in Europe, taking into account data from other Europeans cohorts (German, Italian, Dutch and Spanish (Table 1) [ 11, 12, 15, 17]. Our results are more comparable with data from Jewish, Cyprus, Serbian and Northern Finnish population [7, 9, 13, 18]. However, this data may be significantly underestimated, due to the limited number of patients included in the studies, as well as the small number of comparative data from other cohorts in the world. A lower percentage of diagnosed SCA27B cases has been described only in East Asia (ex. Japan, China), which may be related to the specificity of the region and the low frequency of alleles ≥ 250 GAA in the Japanese and Chinese population compared to the Caucasian population, which is explained by different polymorphisms [19, 20]. Data from European cohorts indicates that SCA27B is a common cause of autosomal dominant ataxia and points for the need of implementation of this type of SCA into the basic diagnostic protocol [1, 9, 11, 12, 13]. The occurrence of episodic symptoms, the late age of onset, cerebellar ocular motor signs and the slowly progressive course of the disease should trigger evaluation for SCA27B [2]. Vestibular hypofunction has also been observed, often with dizziness and loss of balance [2, 5]. The occurrence of rare cases of young adults (≥ 25 years) with SCA27B indicates that the diagnosis should also be considered in younger individuals with a suggestive phenotype and an otherwise negative genetic workup [5, 8, 9].The spectrum of symptoms observed in the cohort of Polish patients—with a predominance of balance disturbances, gait ataxia, and cerebellar syndrome, along with the presence of episodic symptoms and dysarthria corresponds to the clinical phenotype of SCA27B described in other cohorts worldwide [1, 6, 7, 9, 11, 13–18]. As a limitation, no data on the presence of downbeat nystagmus, a hallmark feature of SCA27B [1, 2, 6, 8, 9, 11,], was available in our cohort.

Accumulating evidence indicates that the clinical penetrance of *FGF14* GAA·TTC repeat expansions is strongly dependent on repeat length. Expansions in the 250–299 GAA·TTC range are considered pathogenic but show incomplete and age-dependent penetrance, as asymptomatic carriers have been reported across multiple populations, including elderly individuals, and a substantial proportion of affected patients present as apparently sporadic cases [2, 5, 7, 8, 11]. In contrast, expansions of ≥ 300 GAA·TTC repeats are regarded as highly penetrant and are associated with a characteristic SCA27B phenotype in the vast majority of reported carriers; however, complete penetrance has not been formally demonstrated, and rare asymptomatic carriers with large expansions have been described, suggesting possible age-dependent or modifying effects [2, 5, 7, 15]. Across cohorts, a wide variability in age at onset has been reported, with symptom onset ranging from early adulthood to late life, and no consistent cerrelation between repeat length ant age at onset has been established [2, 5, 6, 11, 17]. This heterogeneity may reflect biological factors such as somatic repeat instability, tissue mosaicism, and genetic or epigenetic modifiers influencing disease expression beyond repeat length alone [2, 3, 5, 15]. Methodological differences (including the definition of age at onset and technical variability in repeat length estimation), relatively small cohort sizes and a limited range of repeat lengths may further reduce the power to detect genotype- phenotype associations [14, 15, 20, 21].

Data on patients with biallelic *FGF14* repeat expansions are currently scarce. Available reports indicate that individuals carrying expansions on both alleles do not appear to present with markedly earlier disease onset or a more severe clinical phenotype compared with heterozygous carriers of a pathogenic expansion [3, 5, 15]. However, due to the limited number of reported cases, larger cohorts and longitudinal clinical studies are required to better define potential- genotype- phenotype correlations in SCA27B [3, 5, 15, 20]. In this context, the interpretation of intermediate alleles remains particularly relevant. Current data indicate that the intermediate allele (180–249 GAA·TTC repeat range) should not be regarded as biologically neutral in the context of SCA27B [15, 21]. Cohort studies have shown that alleles in the 200–249 range are significantly enriched among patients with phenotypes within the SCA27B spectrum (e.g. DBN) compared with controls [21]. Importantly, long-read sequencing studies demonstrate that clinical relevance depends not only on repeat length but also on repeat structure: uninterrupted (,,pure’’) expansions ≥ 200 repeats are associated with disease, whereas interrupted configurations do not show a comparable association [15, 19]. Moreover, one study reports confirmed SCA27B cases in patients carrying at least one allele within the 180–249 GAA repeat range [15]. Taken together, the available evidence suggests that 180–249 GAA·TTC alleles (particularly uninterrupted expansions) should be interpreted as variants with potential clinical significance. These observations support the notion that alleles in the 180–249 repeat range may represent a reduced penetrance category rather than biologically neutral variants [15, 21]. Their assessment should incorporate sequence structure, patient phenotype, and family segregation analysis. Such an approach more accurately reflects the biological spectrum of the disease than a dichotomous classification based solely on the 250- repeat threshold. Our four reported cases with intermediate GAA·TTC alleles (9/224, 9/232, 100/237 and 38/244 GAA·TTC) are consistent with this interpretation and provide additional support for reconsidering the pathogenicity threshold. Together, these findings highlight the complexity of genotype–phenotype relationships in *FGF14*-related ataxia and underscore the importance of interpreting repeat expansion results in the clinical context. They further support the inclusion of *FGF14* repeat expansion analysis in the diagnostic workup of unexplained adult-onset cerebellar ataxia, particularly after exclusion of other genetic and acquired causes [2, 4, 5].

Neuroimaging in SCA27B usually shows mild to moderate cerebellar atrophy, especially in the vermis [1, 2, 5]. In 11/31 of our patients with SCA27B and available MRI results, we observed cerebellar atrophy in 5/11 (45%) which is a common hallmark of SCA27B [14]. Cerebral atrophy was observed in 2/11 cases (18%), suggesting that this change is either uncommon or may appear in later stages of the disease. However, changes of this type are also observed in many other types of SCA, so they are not specific for SCA27B. Moreover, in our patients we did not observe any signs of hyperintensity in the superior cerebellar peduncles (SCP), which have been observed in different cohorts, but FLAIR T2-weighted images were not available for review [11].

To date, this is the only epidemiological investigation of SCA27B in a large cohort of Polish patients with late-onset, undiagnosed ataxia. Although rarer than in other European cohorts, these findings support the inclusion of this form of ataxia in routine diagnostics. However, our study has several important limitations. It was retrospective in nature, and the patients were recruited from across Poland, which often meant very limited access to detailed medical records. Due to the lack of access to detailed MRI images and reports, a comparative analysis of sporadic and familial SCA27B cases is not possible.The lack of SARA scale scores for patients with confirmed SCA27B, as well as the absence of neuroimaging data for more than half of the patients, makes it impossible to assess the long-term progression of the disease. Additionally, it was not possible to perform segregation studies in any of the families. More detailed studies are needed to thoroughly characterize the clinical phenotype of SCA27B and the frequency of mutations in the range of incomplete and full penetrance of the *FGF14* gene in the cohort of Polish patients.

## Electronic supplementary material

Below is the link to the electronic supplementary material.

Supplementary figure 1 (PNG 314 KB)

High Resolution Image (1.08 MB)

Supplementary figure 2 (PNG 139 KB)

High Resolution Image (83.3 KB)

Supplementary figure 3 (PNG 95.3 KB)

High Resolution Image (64.2 KB)

Supplementary figure 4 (PNG 107 KB)

High Resolution Image (119 KB)

Supplementary figure 5 (PNG 167 KB)

High Resolution Image (235 KB)

Supplementary figure 6 (PNG 266 KB)

High Resolution Image (262 KB)

## Data Availability

The datasets generated during and/or analyzed during the current study are not publicly available due to institutional policy restricting the sharing of sensitive clinical and genetic data. This policy is in place to ensure compliance with ethical standards, informed consent limitations, and applicable data protection regulations, including the national General Data Protection Regulation (GDPR) and relevant national laws. However, the data is available from the corresponding author on reasonable request.

## Declarations

### Consents to Participate

Informed consent was obtained from all individual participants included in the study.
