# Supplementary figures and images for: Identification of FGF14 GAA Expansions in Polish Patients with Undiagnosed Cerebellar Ataxia – A Preliminary Study
Source: Cerebellum. 2026 May 7;25(3):73. doi: 10.1007/s12311-026-02003-4 (PMC13152904; doi:10.1007/s12311-026-02003-4)

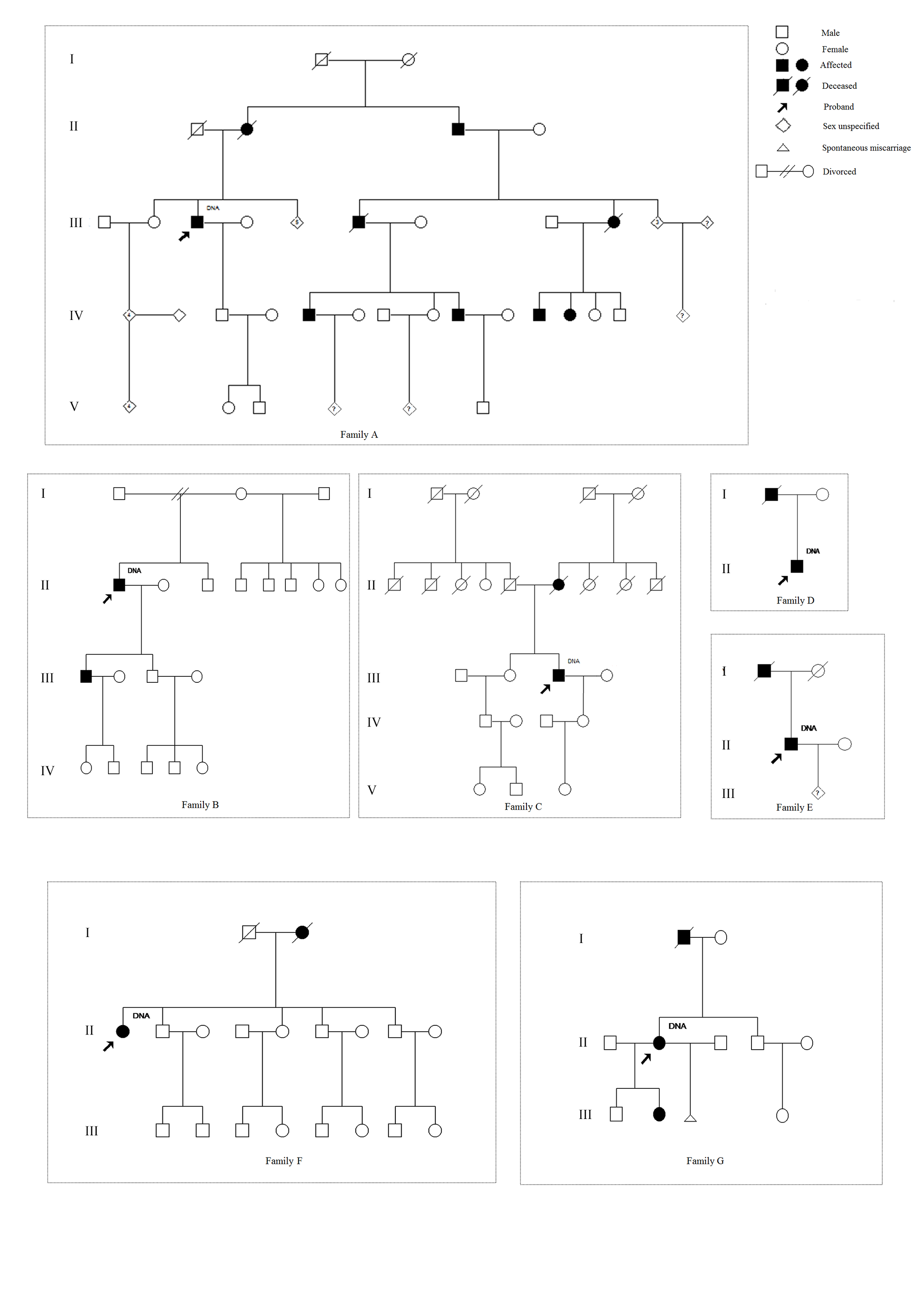

Supplement: Supplementary file 1 — Supplementary figure 1 (PNG 314 KB) [file 12311_2026_2003_MOESM1_ESM.png]

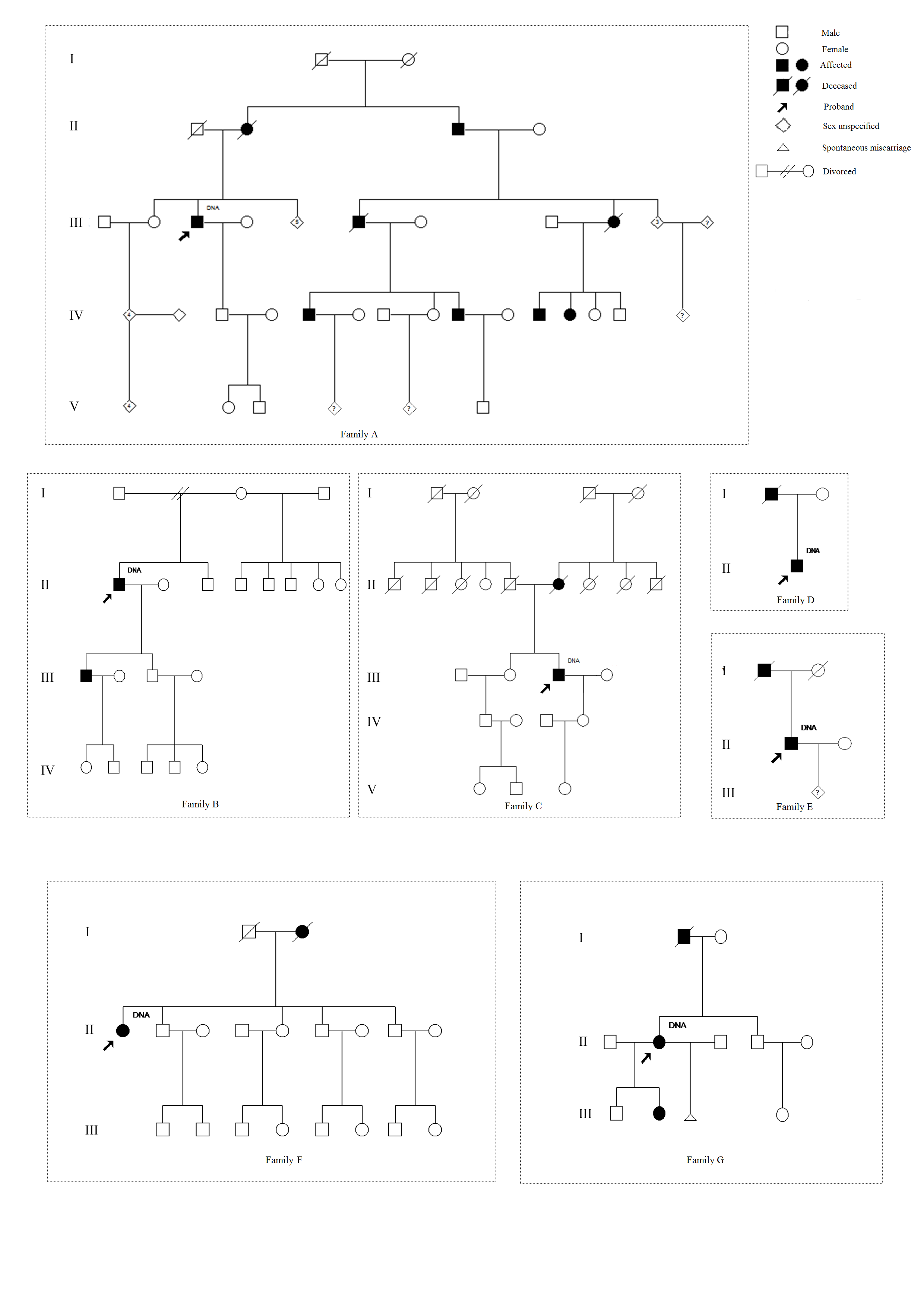

Supplement: Supplementary file 2 — High Resolution Image (1.08 MB) [file 12311_2026_2003_MOESM2_ESM.tif]

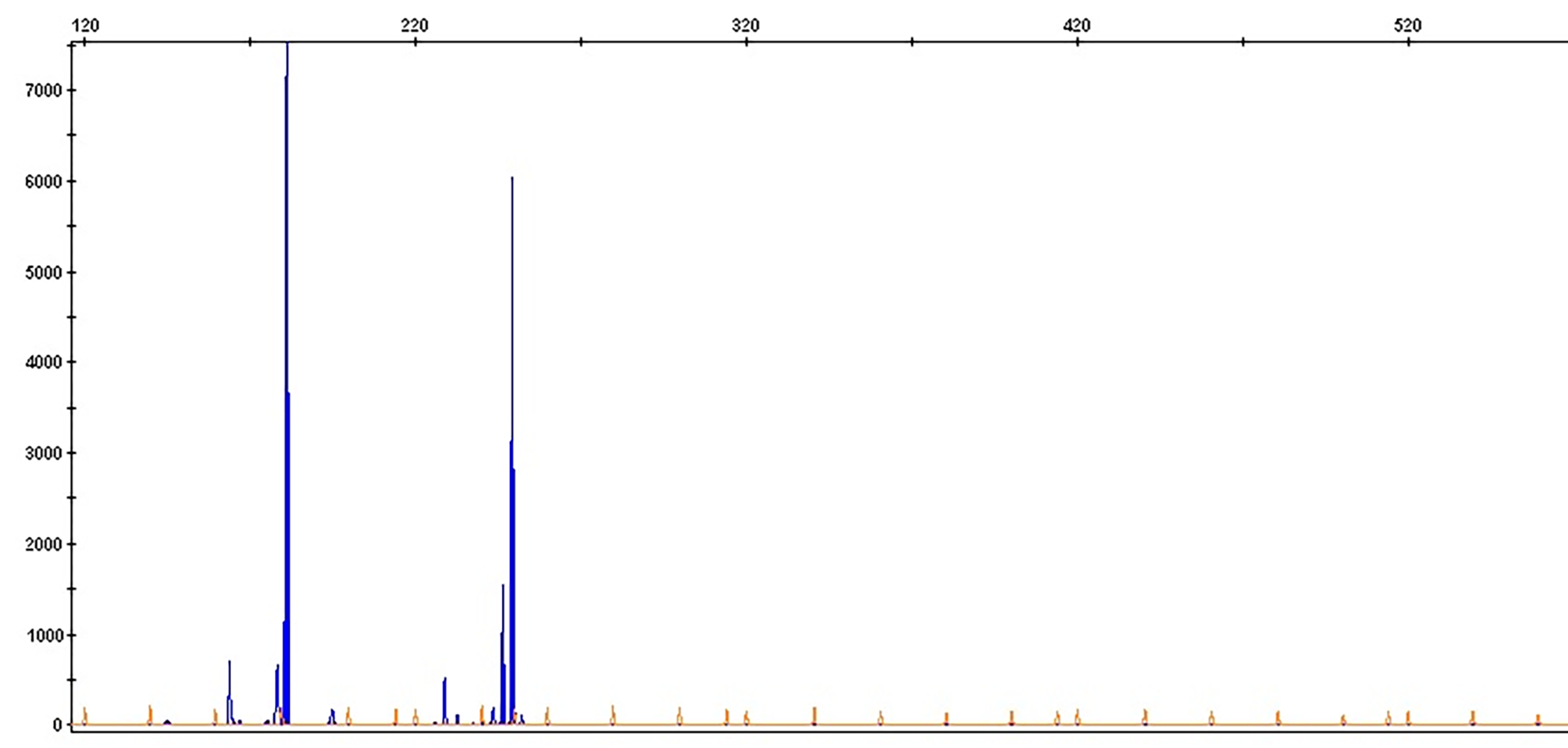

Supplement: Supplementary file 3 — Supplementary figure 2 (PNG 139 KB) [file 12311_2026_2003_MOESM3_ESM.png]

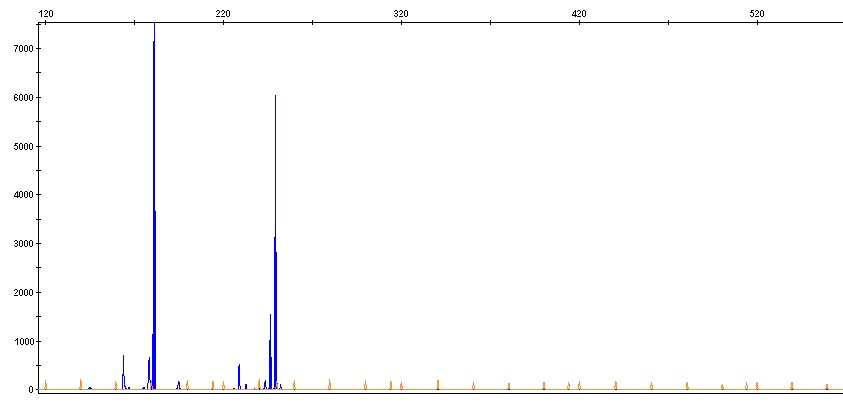

Supplement: Supplementary file 4 — High Resolution Image (83.3 KB) [file 12311_2026_2003_MOESM4_ESM.tif]

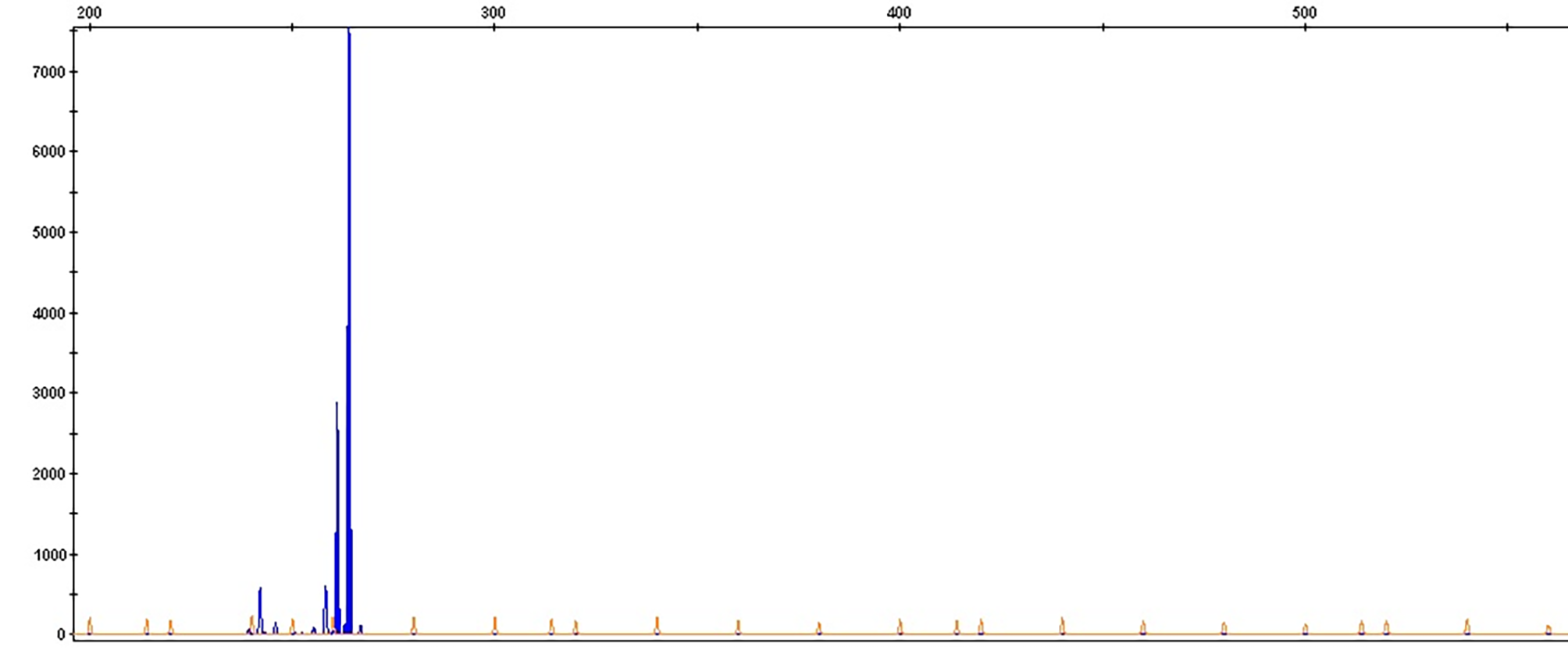

Supplement: Supplementary file 5 — Supplementary figure 3 (PNG 95.3 KB) [file 12311_2026_2003_MOESM5_ESM.png]

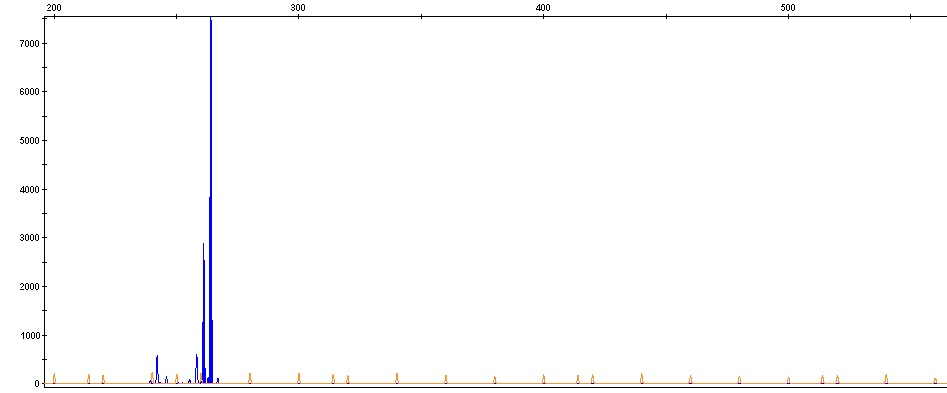

Supplement: Supplementary file 6 — High Resolution Image (64.2 KB) [file 12311_2026_2003_MOESM6_ESM.tif]

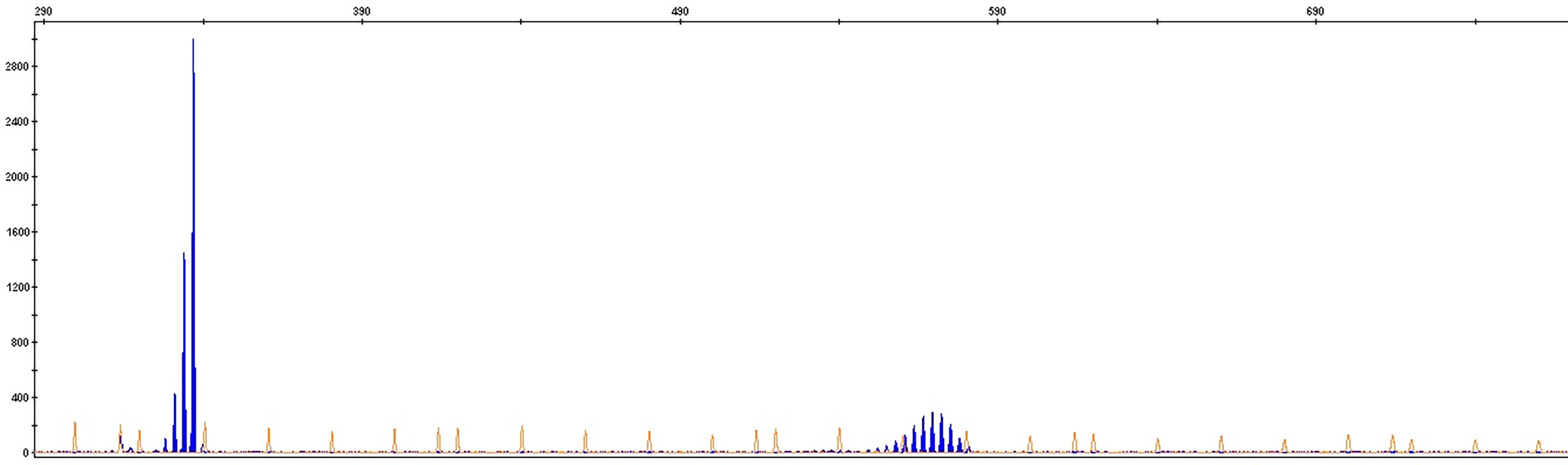

Supplement: Supplementary file 7 — Supplementary figure 4 (PNG 107 KB) [file 12311_2026_2003_MOESM7_ESM.png]

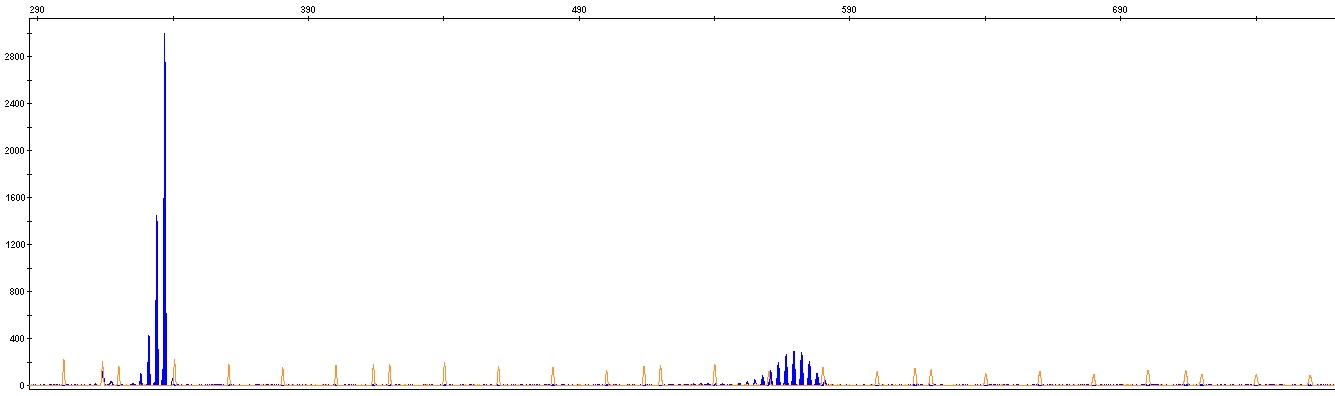

Supplement: Supplementary file 8 — High Resolution Image (119 KB) [file 12311_2026_2003_MOESM8_ESM.tif]

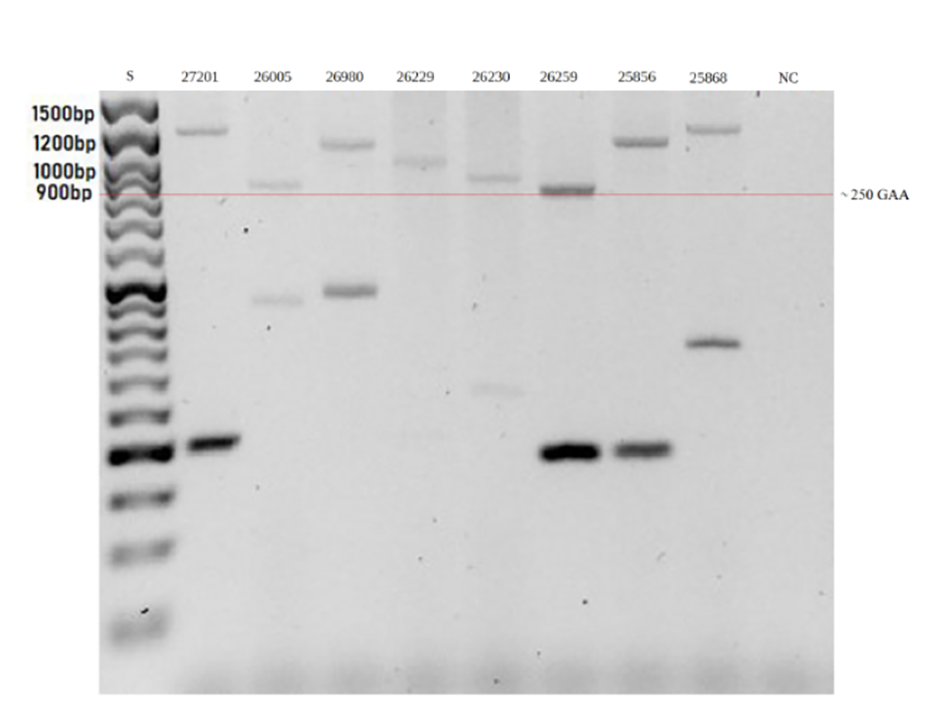

Supplement: Supplementary file 9 — Supplementary figure 5 (PNG 167 KB) [file 12311_2026_2003_MOESM9_ESM.png]

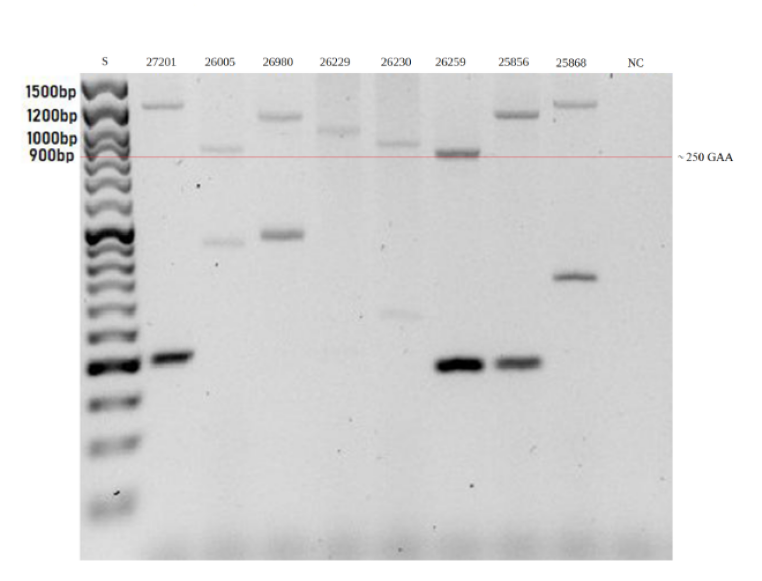

Supplement: Supplementary file 10 — High Resolution Image (235 KB) [file 12311_2026_2003_MOESM10_ESM.tif]

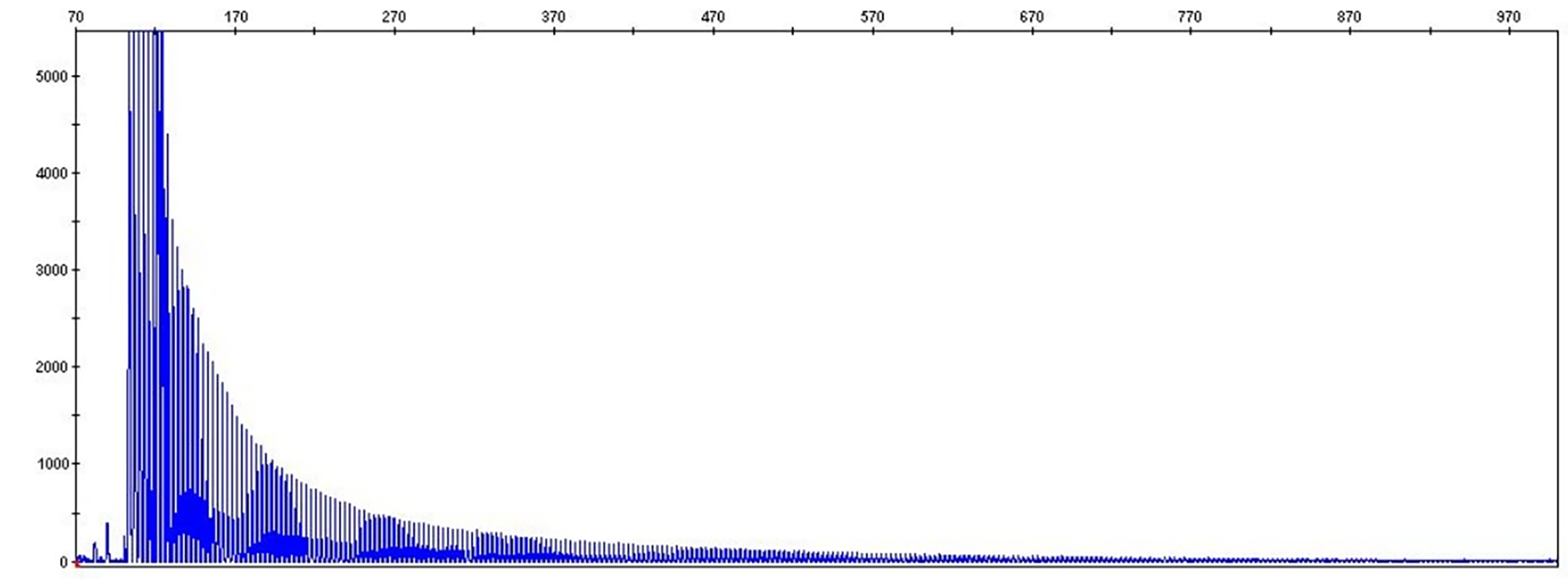

Supplement: Supplementary file 11 — Supplementary figure 6 (PNG 266 KB) [file 12311_2026_2003_MOESM11_ESM.png]

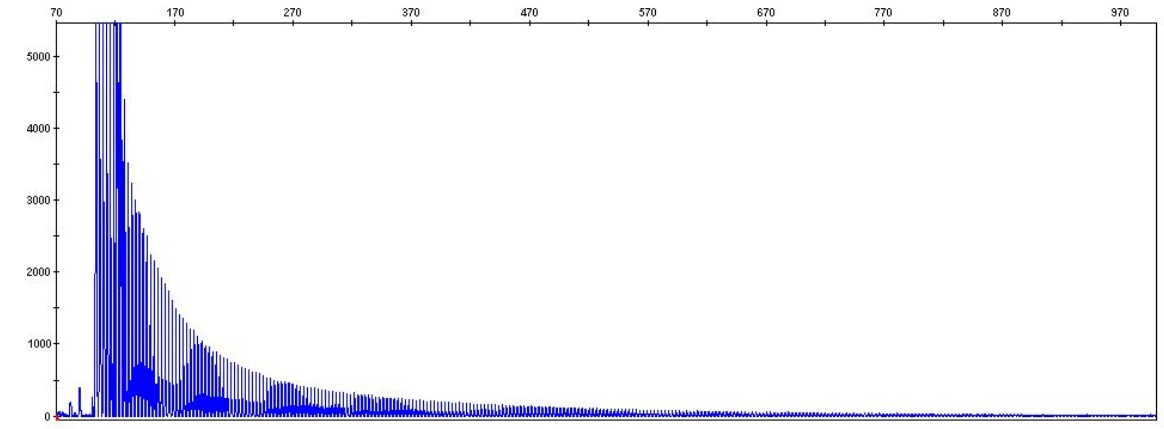

Supplement: Supplementary file 12 — High Resolution Image (262 KB) [file 12311_2026_2003_MOESM12_ESM.tif]
